# Mainstreaming genomic testing for mitochondrial disease in Australia

**DOI:** 10.1038/s41431-026-02053-6

**Published:** 2026-02-26

**Authors:** Megan Ball, Naomi Baker, Sze Chern Lim, Sarah Casauria, Sebastian Lunke, Alison G. Compton, David R. Thorburn, John Christodoulou, Zornitza Stark

**Affiliations:** 1 Murdoch Children’s Research Institute, Melbourne, VIC Australia; 2 Department of Paediatrics, University of Melbourne, Melbourne, VIC Australia; 3 Royal Children’s Hospital, Melbourne, VIC Australia; 4 Victorian Clinical Genetics Services, Murdoch Children’s Research Institute, Melbourne, VIC Australia; 5 Department of Pathology, University of Melbourne, Melbourne, VIC Australia

**Keywords:** Genetics research, Genetic testing

## Abstract

Genomic sequencing has transformed the diagnostic approach for mitochondrial disease, yet integration into standard clinical practice is limited by access and funding. We conducted a post-implementation evaluation of genome sequencing (GS) for mitochondrial disease in Australia, which became publicly funded through the Medicare Benefits Scheme (MBS) in November 2023, to allow for broader access to testing. Test request data, including demographics, phenotypic information, and the diagnostic outcomes, were collected from November 2023 to May 2025 from the Victorian Clinical Genetics Services, the current laboratory provider of the MBS-funded service. Test uptake was 26% of predicted, with lower test rates in regional and remote areas. Over the first 19 months, 300 individuals suspected of mitochondrial disease underwent GS with a median turnaround time of 84 days (8 days–218 days). The diagnostic yield was 20%, with 56% of diagnoses in known mitochondrial disease genes. Of these, 70% (24 of 34) were in mitochondrial DNA. Seventeen diagnoses were in individuals who had prior non-diagnostic testing (exome sequencing or gene panel). We demonstrate that publicly-funded GS can deliver meaningful diagnostic outcomes for mitochondrial disease on a national scale. To maximise its impact, attention must now shift towards ensuring equitable access, particularly for regional and remote areas, and embedding sustainable mainstreaming models that support both genetic and non-genetic clinicians.

## Introduction

Mitochondrial diseases are among the most common inborn errors of metabolism, with an estimated birth prevalence of approximately 1 in 5000 [1]. Mitochondrial diseases are genetically and clinically complex, posing a diagnostic challenge to clinicians. Diagnosis is often delayed, averaging nearly 10 years from symptom onset to diagnosis, substantially greater than rare disease in general [2]. Early diagnosis is critical, enabling timely access to specialist healthcare, connection with community support groups and other families with lived experience [3], and for some individuals, access to targeted therapeutic interventions or clinical trials. Molecular diagnosis facilitates prognostication and can provide families with accurate reproductive counselling and options.

Genomic sequencing has transformed the diagnostic pathway, often recommended now as first-line testing, offering a less invasive and more comprehensive approach than traditional diagnostic tests [4]. Its utility in mitochondrial disease is well-established, with diagnostic yields of 30–70% reported across cohorts, shortening the diagnostic odyssey for families [5]. Despite these advances, equitable access to genomic testing in Australia has been limited by a lack of public reimbursement, regional inequities in access to specialist genetic services, and variable clinician familiarity with testing modalities and pathways. The demand for genomic testing for mitochondrial disease has steadily risen, yet the size of the clinical genetics workforce has not kept pace, further stretching capacity [6]. Traditionally confined to clinical genetics, genomic testing is now increasingly accessed through other non-genetic specialties such as neurology and paediatrics. This shift, often described as ‘mainstreaming’, allows earlier and broader access to testing [7]. Models of mainstreaming vary across different health systems and specialties [7], but the Australian experience has demonstrated that non-genetic clinicians can order and manage tests effectively when supported by clear pathways, education and collaboration with geneticists and genetic counsellors [8, 9].

In 2023, publicly funded genomic testing for mitochondrial disease became available in Australia through the Medicare Benefits Scheme (MBS) following a health technology assessment. However, this pathway represents only one component of a broader diagnostic landscape. The complexity of mitochondrial disease poses unique challenges for implementation in comparison to more narrowly defined clinical indications for testing [7]. Individuals with mitochondrial disease can present with ‘any symptom, in any organ or tissue and at any age’ [10], and have phenotypic overlap with non-mitochondrial disease [1]. Many children and some adults will present with severe or rapidly progressive disease requiring admission to intensive care units, where rapid genomic sequencing may be accessed through alternate funding pathways [11]. This clinical heterogeneity, in combination with the large number of nuclear and mitochondrial encoded genes involved in mitochondrial disease and the unpredictable nature of tissue heteroplasmy in mitochondrial DNA (mtDNA) disorders, presents challenges in the test selection, result interpretation, and counselling for the non-genetic clinician. It is therefore uncertain how mainstreaming models will operate in this setting and what level of involvement from genetic or metabolic services will be needed at different stages of the testing pathway. Ensuring that mainstreaming is equitable and sustainable for mitochondrial disease will require workforce planning, education for non-genetic clinicians, and deliberative service design that fosters collaboration between genetic clinicians and non-geneticists.

Post-implementation audit of publicly funded genomic testing is essential to ensure that tests are being used appropriately and to identify inequities in access. This provides vital evidence for future policy decisions, ensuring that genomic testing remains clinically useful, cost-effective, and responsive to patient needs. Here we report the utilisation and outcomes of genomic testing for mitochondrial disease following its implementation in a national health system.

## Materials and methods

### Study design

We conducted a post-implementation study of the first 300 individuals who underwent publicly funded genome sequencing for suspected mitochondrial disease in Australia from November 2023 to May 2025.

### Context

In Australia, selected genomic tests are publicly subsidised through the MBS, following health technology evaluation and recommendation by the Medical Services Advisory Committee (MSAC). Following the MSAC evaluation for genomic testing for mitochondrial disease, several new MBS-funded tests became available in November 2023. Children and adults are eligible for publicly funded genomic testing if the referring specialist or consultant physician (including genetic and non-genetic specialists) has a high clinical suspicion of a mitochondrial disease and the individual meets one or more predefined clinical features of mitochondrial disease (e.g. unexplained hypotonia or weakness, refractory or atypical seizures, cardiomyopathy, stroke-like episodes or external ophthalmoplegia). Full eligibility criteria can be reviewed in Supplementary Material 1 [12, 13]. Referring specialists can request either genome sequencing (GS) or exome sequencing (ES) with concurrent mtDNA sequencing. Both singleton and trio testing are available, with trio sequencing performed when biological parental samples are available. At the time of implementation, the Victorian Clinical Genetics Services (VCGS) in Melbourne, Australia, was the sole laboratory providing MBS-funded GS for mitochondrial disease. Samples were accepted from referring clinicians nationwide and tests were requested through the VCGS online ordering system. Reports were returned to the referring clinician, with local pathways determining subsequent genetics involvement. In its evaluation, MSAC predicted that 400 adults and 52 children nationally would be eligible to receive GS or ES for suspected mitochondrial disorders within the first year of listing the Medicare item numbers [14].

### Sequencing, data analysis and interpretation

Genome sequencing data generation and clinical analysis were performed using diagnostically accredited methods by VCGS in Melbourne, Australia. GS was performed to enable simultaneous analysis of both the nuclear and mitochondrial genomes. Generally, the mitochondrial genome was analysed first, followed by analysis of the nuclear genome. Variant prioritisation for the nuclear genome followed a sequential approach of phenotype-driven virtual gene panels, progressing to gene-agnostic analysis for trio cases where no diagnosis was reached. DNA was extracted from peripheral blood or muscle using standard methods. Genome sequencing was performed using massively parallel sequencing (Illumina DNA PCR-Free Library Prep kit, Illumina Sequencers) with a mean target coverage of 27x and a minimum of 97.5% of bases sequenced to at least 10x for nuclear DNA, and a minimum of 800x mean coverage for mtDNA. Data were processed using accredited pipelines, including read alignment to the reference nuclear genome (UCSC GRCh38/hg38) and the revised Cambridge Reference Sequence mitochondrial genome (NC_012920.1). Variant calling for nuclear DNA was performed using the Illumina Dragen System. For mtDNA, variants were called using Mutect2. For mtDNA analysis, the assay was clinically validated to reliably detect single-nucleotide variants with heteroplasmy >3% and large-scale mtDNA deletions with heteroplasmy >30%.

Variant prioritisation was performed with analysis of copy number variants (CNVs), intronic variants and the mitochondrial genome as standard [15, 16]. For nuclear DNA, variant analysis and interpretation within the selected target region (RefSeq genes ± 1 kb) was performed using Agilent Alissa Interpret software and later Illumina Emedgene software. CNVs were screened using an in-house CNV detection tool, CXGo [15]. For mtDNA, a custom in-house analysis pipeline was used to detect large deletions and annotate the VCF file with variant information [16].

In addition to the virtual gene panel ‘Mitochondrial disease’(https://panelapp-aus.org/panels/203/), other virtual gene panels were applied by VCGS based on the clinical phenotype provided by the referring clinician. Individuals who had trio genome sequencing (GS) underwent Mendeliome (genes known to be associated with monogenic disease) analysis. If this did not yield a diagnosis, gene-agnostic analysis was performed to identify plausible novel gene candidates. All panels used in the study are publicly available from PanelApp Australia(https://panelapp-aus.org/). Variants were classified using the principles outlined by the American College of Medical Genetics and Genomics [17, 18].

### Data analysis

Electronic test ordering and reports were used to collect demographic, phenotypic data and diagnostic outcomes. Phenotypic information was collected using Human Phenotype Ontology [19] terms. Actual utilisation data were extracted from Services Australia [20] between November 2023 and October 2024. Data were managed using REDCap [21]. Statistical analyses were performed using R [22]. Descriptive statistics were used for individual characteristics, phenotype and diagnostic outcomes. Pearson’s Chi-squared test with Yates continuity correction was used to compare the number of diagnoses in children and adults, between singleton and trios, and between those with a suspicion of a specific diagnosis or not. A pairwise comparison using Wilcoxon rank-sum tests with continuity correction was used to determine if the age of the individual differed between those with a mitochondrial diagnosis, non-mitochondrial diagnosis and those who remained unsolved, and to compare turnaround times between singleton and trio testing. A Fisher’s exact test was used to determine if the diagnostic yield differed between referring specialties and to compare the most common clustered HPO terms between diagnosis groups, with p-values adjusted using the Benjamini–Hochberg false discovery rate method. Postcode data were aggregated to calculate the number of tests undertaken within a Local Government Area (LGA) [23]. The geographical distribution of testing across areas of remoteness according to the Accessibility and Remoteness Index of Australia (ARIA + ) and socioeconomic status according to the Socio-Economic Index for Areas (SEIFA) was determined using a previously described method [24]. The relationship between the number of tests and remoteness category or SEIFA quintiles was determined by Spearman’s rank correlation coefficient.

## Results

### Demographics

**Fig. 1:**
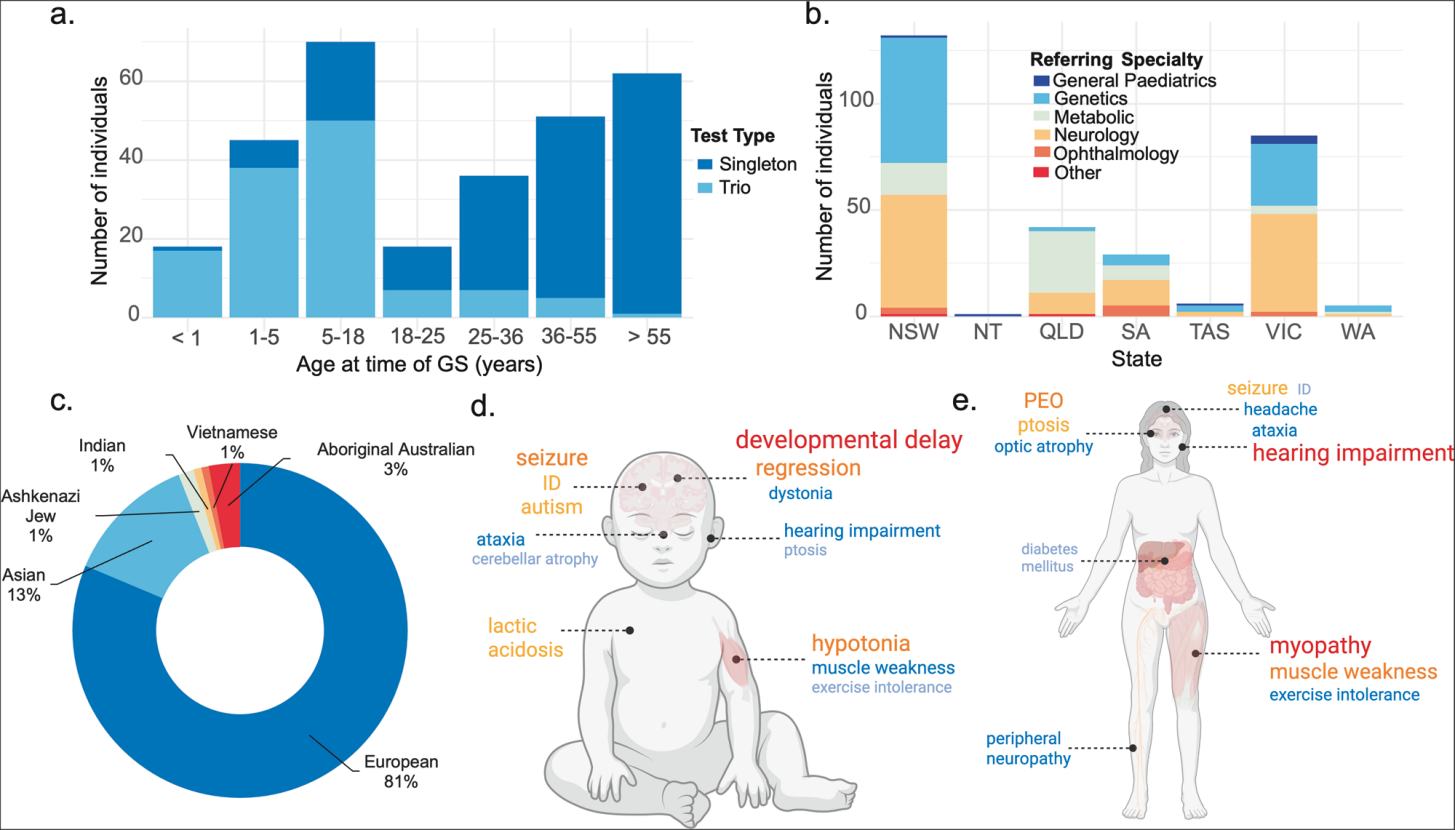
Demographics and phenotypic features of individuals who underwent publicly funded testing for mitochondrial disease. **a** Age at time of testing and test type. **b** Number of individuals referred for testing in each state, split by referring specialty. **c** Ancestry of 134 individuals with reported ancestry (**d**). Common phenotypic terms for children, Size of each phenotypic term representative of number of individuals affected. **e** Common phenotypic terms for adults. Size of each phenotypic term representative of number of individuals affected. GS Genome sequencing, NSW New South Wales, NT Northern Territory, QLD Queensland, SA South Australia, TAS Tasmania, VIC Victoria, WA Western Australia, PEO progressive external ophthalmoplegia, ID intellectual disability. Created in BioRender. Christodoulou, J. (2025) https://BioRender.com/ogr2356 and https://BioRender.com/5ce0ngr.

We analysed the first 300 individuals to undergo publicly funded genomic testing for suspected mitochondrial disease in Australia between November 2023 and May 2025. The cohort included 159 (53%) females and 167 (56%) adults. Age at time of testing spanned from 3 months to 96 years, with a median age of 25 years (IQR 7–50) (Fig. 1a). Trio analysis was performed for 125 (42%) families and singleton GS was performed for 175 (58%) individuals. DNA was extracted from blood for 88% of individuals, with 5% extracted from muscle, and the remainder using DNA extracted externally. The median turnaround time (TAT) was 84 days (IQR 67–104). Singleton GS had a longer median TAT (98 days), while trio GS was comparatively faster (67 days)(*p* < 0.001). The majority of referring clinicians were neurologists (41%), clinical geneticists (34%) and metabolic clinicians (19%) (Fig. 1b). Information provided by the clinician about ancestry was only available for 134 individuals and disproportionately represented individuals of European descent (81% of reported ancestry)(Fig. 1c).

**Fig. 2:**
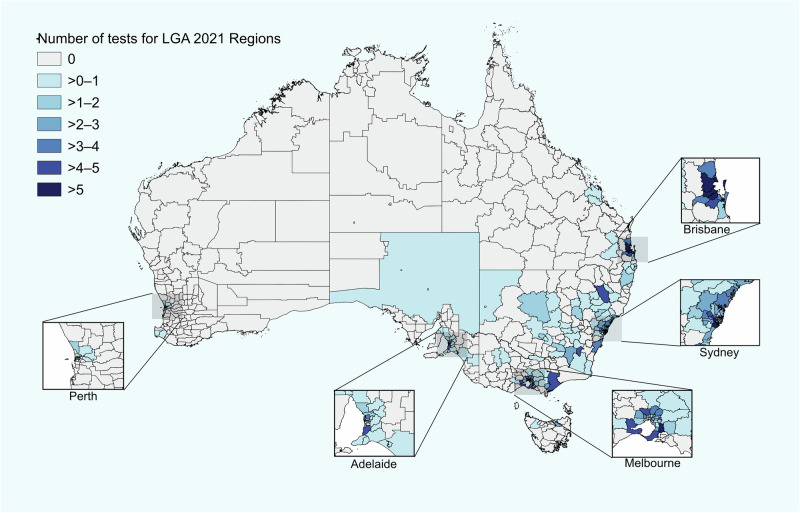
Geographical distribution of genomic testing in Australia by local government area (LGA). Map of Australia displaying the number of tests per LGA, showing higher uptake in major cities, which recorded a higher number of tests relative to regional, remote, and very remote areas.

**Fig. 3:**
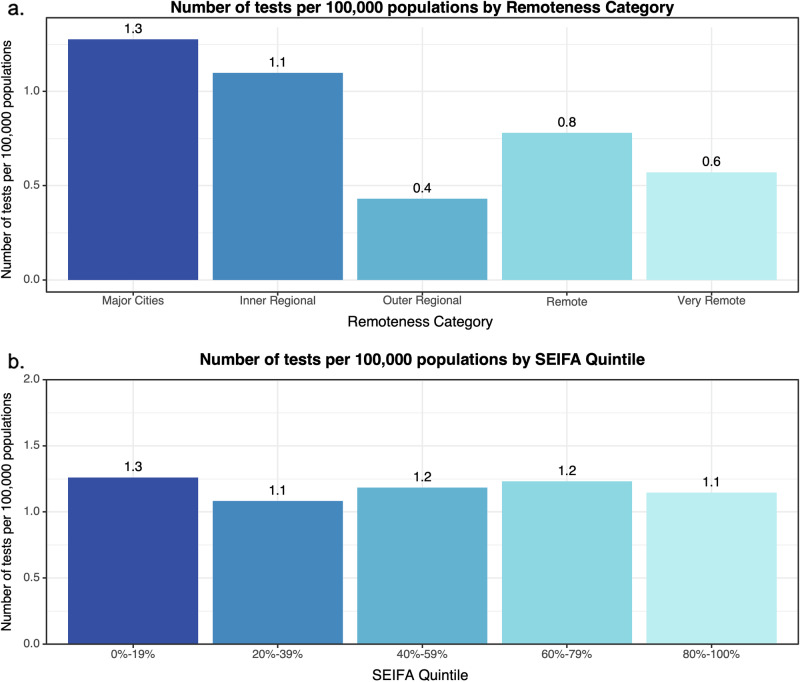
Genomic testing rates per 100,000 population by remoteness and socioeconomic status. Number of tests per 100,000 population across (**a**). Remoteness Categories (Major Cities to Very Remote) and (**b**). Socio-Economic Indexes for Areas (SEIFA) quintiles (0-19% most disadvantaged to 80-100% least disadvantaged).

The largest proportion of individuals were from New South Wales (42%)(Fig. 1b). The median distance for the individual to travel via road to the referring clinician was 28 km (IQR 13–90). There was a significant difference in the number of genomic tests per LGA across Remoteness Categories with LGAs in Major Cities (*n* = 239, 80%) having more tests than those in Inner Regional (*n* = 48, 16%), Outer Regional (*n* = 8, 2.6%), Remote (*n* = 2, 0.7%), and Very Remote (*n* = 1, 0.3%) areas (adjusted *p* < 0.001)(Fig. 2). Spearman’s rank correlation coefficient identified a negative relationship between geographic remoteness and the number of tests performed per LGA (rho = -0.53, *p* < 0.0001), consistent with the distribution of the Australian population. However, when corrected for population size, the rate of testing was still lower in more remote regions, suggesting inequity in access based on geographic remoteness (Fig. 3). The number of tests was significantly higher in the most socioeconomically advantaged LGAs (80–100% quintile) compared to all other quintiles except the 60–79% quintile (adjusted *p* < 0.001-0.01). While there was a moderate positive correlation between SEIFA percentile and the number of genomic tests per LGA (rho=0.28, *p* < 0.0001), this relationship did not persist when rates were normalised by population size. The number of tests per 100,000 population did not differ across SEIFA quintiles (Fig. 3), suggesting that, at a population level, access to testing was relatively equitable across socioeconomic groups.

### Uptake

Within the first year of testing (November 2023-October 2024), 116 individuals (69 adults, 47 children under the age of 15) underwent genomic testing, which was 26% of the predicted. Of those tests, 56% (65/116) were singletons and 44% (51/116) were trios. However, rates of testing increased over the study period (Supplementary material 2), with the number of tests reported in April and May 2025 approaching the predicted rates (~38 individuals per month).

### Phenotype

The range of phenotypes recorded was broad, ranging from severe multisystem disease to isolated system involvement (e.g. optic atrophy or diabetes mellitus). The median number of HPO terms per individual was 5 (range 1–20). Most individuals (*n* = 217) had nervous system involvement and the most common HPO term was HP:0001250: seizure (*n* = 50). Individuals often met multiple testing criteria, with the most common criterion met ‘ataxia, encephalopathy, seizures, muscle fatigue or weakness’ (*n* = 171). HPO terms were clustered based on common ontology hierarchy. Developmental delay, developmental regression, and/or seizures were the HPO terms most commonly seen together. Progressive external ophthalmoplegia (PEO), peripheral neuropathy, and myopathy were more likely to be reported in adults compared to children (*p* < 0.001), while conversely global developmental delay, regression and hypotonia were more likely to be reported in children than adults (*p* < 0.001) (Fig. 1d, e).

### Diagnostic yield

Of the 300 individuals tested, 61 (20%) received a definitive primary diagnosis. Of those, 34 (56%) were in mitochondrial disease genes (Supplementary table 1) and 24 (10 children, 14 adults) were mtDNA in origin. Seven of those individuals had the well-reported pathogenic mtDNA variant, NC_012920.1(*MT-TL1*):m.3243 A > G (age-adjusted heteroplasmy ranging from 24-93% in blood and 76% heteroplasmy in muscle in one individual). Ten diagnoses (three children, seven adults) were made in the nuclear-encoded mitochondrial disease genes (Supplementary Table 1).

A non-mitochondrial diagnosis was made in 27 individuals (44%) following phenotype-driven analysis of other clinically relevant virtual panels. Most of these diagnoses were made in genes associated with neurodevelopmental syndromes (59%), with other diagnoses made in genes associated with cardiomyopathy, neurodegenerative disease, deafness, epilepsy, or other inborn errors of metabolism. Five of those individuals received a partial diagnosis that did not explain their entire clinical phenotype. Some of the non-mitochondrial diagnoses were actionable or treatable, including other inborn errors of metabolism, e.g. GLUT1 deficiency (MONDO:0000188) and cancer predisposition syndromes (PTEN syndrome (MONDO:0017623), ataxia-telangiectasia (MIM#208900)). No individual received a dual diagnosis.

Seventeen individuals who received a definitive diagnosis (ten mitochondrial, seven non-mitochondrial) had previously had a non-diagnostic ES, mtDNA sequencing and/or a gene panel. Eight of these diagnoses involved the mitochondrial genome. Two individuals received a diagnosis of a single mtDNA deletion in muscle after both previously having negative ES, with one also having negative mtDNA sequencing in blood. Genome sequencing would have been required to detect the causative variant/s in eight individuals with nuclear diagnoses (mitochondrial and non-mitochondrial), as ES would not reliably identify the variants responsible. An individual with epileptic encephalopathy who had previously had a non-diagnostic trio ES was identified to have an intragenic copy number variant resulting in an out-of-frame deletion of exon 5 of *RARS2* (MIM#611524), which was in trans with a likely pathogenic missense variant resulting in a diagnosis of pontocerebellar hypoplasia, type 6 (MIM#611523). Another individual with intellectual disability, microcephaly and seizures who had previously had a non-diagnostic trio ES was identified to have a complex structural variant involving chromosome regions Xq28 and 7q36.3, with the phenotype suspected to be caused by the insertion of a region from 7q36.3 into the final exon of *MECP2* (MIM#300005) on Xq28 resulting in a diagnosis of Rett syndrome (MIM#312750).

Of the remaining 239 individuals who received a non-diagnostic report, 184 had no variants of uncertain significance (VUS) relevant to their phenotype reported. Nearly 22% of individuals (*n* = 51) with a non-diagnostic report had variants of potential clinical relevance in nuclear or mtDNA disease genes, some of whom have been referred for segregation testing and/or functional studies. Five individuals received incidental findings, including one individual who received a primary diagnosis of Leigh syndrome, *MT-ND1*-related (MONDO:0009723), in addition to an incidental finding of von Willebrand disease, type 2B (MIM#613554).

### Factors affecting diagnostic yield

More children received a diagnosis compared to adults (25% vs 17%), although this was not statistically significant (*p* = 0.12). Children were more likely to undergo trio GS than adults. The diagnostic yield of trio GS (23%) and singleton GS (18%) was not significantly different. When stratified by both age group and test type, diagnostic yield remained higher in children for both singleton and trio analyses, and no independent association between age group or test type and diagnostic outcome was observed. Individuals with non-mitochondrial diagnoses tended to be younger in age than individuals with mitochondrial diagnoses or individuals not receiving a diagnosis (*p* < 0.01 and *p* < 0.001, respectively), whereas there was no clear age difference between individuals with mitochondrial diagnoses and individuals not receiving a diagnosis. The diagnostic yield did not significantly differ by referring specialty, with the clinicians who frequently ordered the test having similar yields (Metabolic physician 23%, Geneticist 21%, Neurologist 18%). Clinicians were more likely to suspect a specific diagnosis in individuals who received a mitochondrial diagnosis (*p* < 0.01) compared to those who received a non-mitochondrial diagnosis or those who remained undiagnosed. This may reflect that some mtDNA disorders have well-defined phenotypes (e.g. Mitochondrial myopathy, encephalopathy, lactic acidosis and stroke-like episodes, Leigh syndrome or Leber hereditary optic neuropathy).

**Fig. 4:**
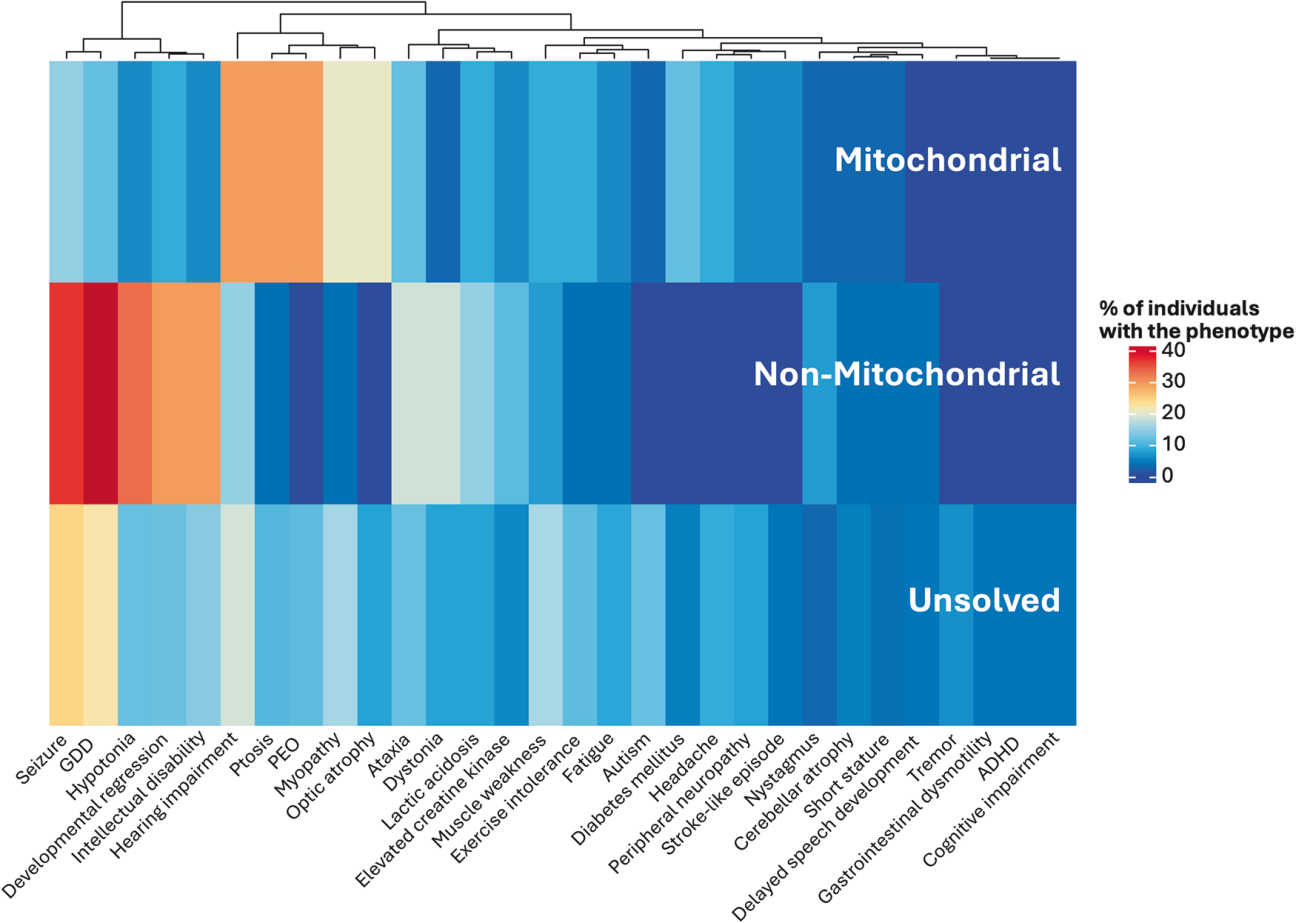
Distrubition and clustering of phenotypic features across diagnostic categories. Heatmap supported by dendrogram depicting the prevalence and clustering of phenotypic features in individuals with a mitochondrial diagnosis, a non-mitochondrial diagnosis or individuals who remain unsolved. GDD global developmental delay, ID intellectual disability, PEO progressive external ophthalmoplegia, ADHD attention deficit hyperactivity disorder.

The most common phenotypic terms for individuals diagnosed with a mitochondrial disease were hearing impairment, PEO and ptosis (29%, 10/34). The most common phenotypic terms for individuals diagnosed with a non-mitochondrial disease were global developmental delay (41%, 11/27) and seizures (37%, 10/27). While there was a trend towards certain phenotypic patterns (Fig. 4) for those who were diagnosed with a mitochondrial disease compared to those who received a non-mitochondrial diagnosis, this was not statistically significant. For example, PEO was observed in 10 adults with a mitochondrial diagnosis and no individuals with a non-mitochondrial diagnosis (adj *p* = 0.09).

## Discussion

We report the national uptake and outcomes of publicly funded genomic testing for mitochondrial disease over the first 19 months of its use, providing data on real-world utilisation to guide future policy and practice. The implementation of genomic testing into healthcare systems should not be judged solely on its diagnostic yield, but on its clinical and personal utility, and whether it is equitable, sustainable, and meets the needs of the population it was designed for [25, 26].

The uptake of testing in the first year was lower than anticipated, reaching only 26% of the predicted activity. This is consistent with another publicly funded genomic test in Australia, which achieved 7% of predicted uptake within its first year [27]. Encouragingly, uptake of testing for mitochondrial disease increased over the study period (Supplementary material 2), possibly reflective of growing awareness among clinicians and advocacy from mitochondrial disease community groups. Diffusion models of genomic test adoption in Australia [28] suggest that mitochondrial disease testing may have benefited from a relatively large pool of early adopters compared to other publicly funded testing, supported by clinician champions and the established research networks in this field. Despite this, our data highlight inequities in access. Individuals from New South Wales and Victoria were over-represented, while those from Western Australia, Northern Territory, and regional and remote areas were under-represented per capita. This may in part be contributed to by different funding models in public health systems, where some clinicians access alternative funding. Lower uptake in regional and remote areas mirrors well-documented challenges in access to genomics across Australia [29]. To address this, services should prioritise culturally safe outreach, telehealth-enabled counselling, and pathways that reduce reliance on urban-based specialists. Without such measures, publicly funded genomic testing risks reinforcing, rather than reducing, existing inequities.

Scaling genomic testing sustainably and equitably requires mainstreaming into standard diagnostic pathways for non-genetic clinicians. Barriers to mainstreaming testing for mitochondrial disease arise at both the individual clinician level (lack of knowledge or confidence) and the system level (geographical access, access to genetics or counselling support, and time) [30, 31]. Lessons from Australian Genomics [32] and other initiatives emphasise that successful mainstreaming depends on clinician champions who drive uptake within their specialties [33]; embedded genetic counsellors[8]; ongoing education across a broad range of specialties [34]; and laboratory capacity with the workforce skills to deliver timely results. Given that many non-genetic clinicians would prefer to refer to genetic clinicians rather than arrange genomic testing [35], embedding these supports into mainstream care is essential to build confidence in test ordering, interpretation, and communication of results. In our cohort, neurologists accounted for 41% of referrals, followed by geneticists (34%) and metabolic physicians (19%), with smaller contributions from general paediatricians and ophthalmologists. This pattern likely reflects early adoption by specialties with established expertise in mitochondrial disease and genomic testing. Importantly, diagnostic yield did not differ significantly by referring clinician, suggesting that genomic testing can be effectively initiated outside specialist genetics clinics when adequate support is available. These data therefore identify both a foundation for implementation and opportunities for targeted expansion, particularly to empower less-represented clinicians, including general paediatricians and those working in rural and remote settings. Multidisciplinary team (MDT) models may provide additional support, though scaling MDTs nationally for mitochondrial disease would be resource-intensive [8]. International experience, including the 100,000 Genomes Project in the UK, emphasises that specialist genomic MDTs facilitate access to care but acknowledge the challenge of staffing such models at scale [36]. A sustainable framework for genomic integration must therefore balance specialist oversight with scalable models that empower non-genetic clinicians, whilst ensuring geneticists continue to play a key role, especially in the interpretation and counselling of complex diagnoses and reproductive counselling, particularly for mtDNA disorders.

The diagnostic yield of this cohort (20%) is lower than that reported in other mitochondrial disease cohorts using GS (31–55%) [11, 37–40]. Direct comparisons are complicated by differences in participant selection and prior testing. Many published cohorts are highly selected, with stringent entry criteria. In contrast, our cohort is heterogeneous in both age and phenotype, with broad eligibility criteria, which is more comparable to the large mitochondrial disease cohort in the 100,000 Genomes Project which reported a 31% diagnostic yield [37]. Notably, almost half of our diagnoses were non-mitochondrial disorders (44%), underlining the phenotypic overlap between mitochondrial disease and its mimics, and the importance of a broad analysis approach. De novo variants accounted for nearly half of the individuals with a non-mitochondrial diagnosis (48%), most often in children with neurodevelopmental syndromes. Importantly, several of the non-mitochondrial diagnoses were actionable or treatable, although our study was not designed to evaluate downstream clinical utility.

The relative proportion of mtDNA vs nuclear mitochondrial diagnoses, especially in children, differed from other recent studies [37, 39, 40]. Paediatric-onset mitochondrial disease is usually associated with nuclear gene variants (80%) [41], whereas only 23% of children in our study had nuclear-encoded mitochondrial disease. In our setting, children who present with severe disease may undergo rapid genomic testing as hospital inpatients, or children may have undergone ES through alternative funding pathways, with those diagnosed not progressing to GS. A limitation of this study is that we are unable to capture testing performed through alternative funding pathways nationally during the study period. In contrast, 67% of adults with mitochondrial disease had mtDNA-related diagnoses, consistent with other studies [5, 38]. We observed many common pathogenic mtDNA SNVs (e.g. m.3242 A > G), which are often under-represented in research cohorts, where such ‘easy’ diagnoses are often excluded [39].

Diagnostic yield was higher in children than in adults, though not statistically significant. Children more often underwent trio sequencing, facilitating detection of de novo variants in non-mitochondrial disease and phasing of variants in recessive disease [42]. However, diagnostic yield remained similar across both singleton and trio testing, indicating that age and test type alone do not fully explain differences in diagnostic outcomes. Nevertheless, diagnoses were identified across the lifespan, with the oldest individual aged 77 years old, emphasising the continuing relevance of genomic testing at all ages. Some adults may have remained undiagnosed due to inappropriate tissue selection for their phenotype. For adults with a highly suspicious phenotype (e.g. PEO), sequencing in post-mitotic tissue should be considered given the possibility of single large-scale mtDNA deletions restricted to skeletal muscle [39] and the decreasing heteroplasmy levels of some mtDNA SNVs in blood over time [43]. We identified single large-scale mtDNA deletions in muscle in two adults with PEO, one of whom had negative mtDNA sequencing in blood.

While the new MBS items allow clinicians to either order GS or ES with mtDNA sequencing, thus far, all individuals have received GS. GS, particularly trio GS, offers several advantages over ES in the diagnosis of rare diseases [42]. GS enables the detection of different classes of variants frequently missed by other methods, including structural variants or rare intronic variants and provides more uniform coverage across coding regions [42, 44]. Although we did not directly compare GS to ES, we identified 17 diagnoses that were not detected on prior sequencing and eight causal nuclear-encoded variants that would have required GS for detection, as they would not have reliably been picked up on ES. GS has been shown to have a diagnostic uplift of 8% in individuals who have undergone previous testing [44] and is increasingly cost-effective as its price approaches that of ES [45].

Prior suspicion of mtDNA syndromes in adults in this study increased the likelihood of identifying the relevant expected mtDNA SNV. This suggests that targeted mtDNA testing may continue to be an appropriate (and cost-effective) choice in adults with specific phenotypes associated with pathogenic mtDNA SNVs (e.g. m.3243 A > G in *MT-TL1* or m.11778 G > A in *MT-ND4*). However, GS should be considered as the first-line test of choice even in phenotypically well-defined cases, as differential diagnoses remain possible e.g. *OPA1* in optic atrophy. Moreover, when pathogenic mtDNA variants are identified at intermediate heteroplasmy levels, the ‘grey zone’, the absence of an alternative diagnosis on GS may help interpret the clinical significance of the mtDNA variant.

In this study, 239 individuals remained without a diagnosis, although nearly 25% had a VUS relevant to their phenotype. Periodic re-analysis of GS/ES data may identify pathogenic variants as new gene-disease associations are discovered [46]. This is relevant in mitochondrial disease, given the discrepancy between the 400 known mitochondrial disease genes and over 1100 known proteins in the mitoproteome [47]. RNA sequencing and proteomics have emerged as powerful tools for mitochondrial disease diagnosis [48, 49]. Recently, the application of untargeted mass-spectrometry proteomics in different tissue types (including peripheral blood mononuclear cells) provided definitive functional evidence for VUS in multiple individuals with mitochondrial disease, obviating the need for invasive muscle biopsy in some [50]. Given the number of individuals suspected of mitochondrial disease that remain unsolved following GS, establishing clear pathways for high-throughput functional assays will be an essential component in the clinical diagnostic pipeline to realise the full potential of genomic-first testing strategies.

This study provides important data on the use of publicly funded GS as first-line testing in a real-world setting for suspected mitochondrial disease. It highlights the utility of broad analysis strategies, and hence the importance of pre-test counselling reflecting the reasonable possibility of a non-mitochondrial diagnosis. There is a clear need for a framework to systematically review the implementation of genomic tests into standard clinical practice, including the definition of what constitutes appropriate outcome measures to drive future policy and practice to ensure equitable, timely and sustainable implementation.

## Supplementary information

Supplementary Tables

Supplementary Material

## Data Availability

The variants identified in this study have been submitted to ClinVar (Submission IDs: SUB15793101, SUB15793127, SUB15796069, SUB15768047). All submitted data are publicly available.
