# Supplementary material for: Mainstreaming genomic testing for mitochondrial disease in Australia
Source: Eur J Hum Genet. 2026 Feb 26;34(5):658–66. doi: 10.1038/s41431-026-02053-6 (PMC13172319; doi:10.1038/s41431-026-02053-6)
Supplement: Supplementary file 2 — Supplementary Material [file 41431_2026_2053_MOESM2_ESM.docx]

**Supplementary Material 1.** Eligibility criteria for publicly funded genomic testing

The Medicare eligibility criteria for publicly funded genomic testing for mitochondrial disease is publicly available from^1,2^. <https://www9.health.gov.au/mbs/fullDisplay.cfm?type=item&q=73456&qt=item&criteria=73456> and <https://www9.health.gov.au/mbs/fullDisplay.cfm?type=item&q=73457>.

Medicare Item No: 73456

Characterisation by whole genome sequencing, or by either or both whole exome sequencing and mitochondrial DNA sequencing, of germline variants present in nuclear DNA and in mitochondrial DNA of a patient with a strong suspicion of a mitochondrial disease, if:

(a) the characterisation is requested by a specialist or consultant physician; and

(b) the characterisation is requested because of the onset of one or more clinical features indicative of mitochondrial disease, including at least one or more of the following:

- I: meeting the clinical criteria of a probable indicator of mitochondrial disease on a relevant scoring system;
- II: evident mitochondrial dysfunction or decompensation;
- III: unexplained hypotonia or weakness, profound hypoglycaemia or “failure to thrive” in the presence of a metabolic acidosis;
- IV: unexplained single or multi-organ dysfunction or fulminant failure (including, but not limited to, neuropathies, myopathies, hepatopathy, pancreatic and/or bone marrow failure);
- V: refractory or atypical seizures, developmental delays or cognitive regression, or progressive encephalopathy or progressive encephalomyopathy;
- VI: cardiomyopathy and/or cardiac arrythmias;
- VII: rapid hearing or painless visual loss or ptosis;
- VIII: stroke-like episodes or nonvasculitic strokes;
- IX: ataxia, encephalopathy, seizures, muscle fatigue or weakness;
- X: external ophthalmoplegia;
- XI: hearing loss, diabetes, unexplained short stature, or endocrinopathy;
- XII: family history of mitochondrial disease, or any of the above; and

(c) the service is not a service associated with a service to which item 73358, 73359 or 73457 applies.

Applicable only once per lifetime

Medicare Item No: 73456

Characterisation by whole genome sequencing, or either or both whole exome sequencing and mitochondrial DNA sequencing, of germline variants present in nuclear DNA and in mitochondrial DNA, of a patient with a strong suspicion of a mitochondrial disease, if:

(a) the characterisation is performed using a sample from the patient and a sample from each of the patient’s biological parents; and

(b) the request for the characterisation states that singleton testing is inappropriate; and

(c) the characterisation is requested by a specialist or consultant physician; and

(d) the characterisation is requested because of the onset of one or more clinical features indicative of mitochondrial disease, including at least one or more of the following:

- I: meeting the clinical criteria of a probable indicator of mitochondrial disease on a relevant scoring system;
- II: evident mitochondrial dysfunction or decompensation;
- III: unexplained hypotonia or weakness, profound hypoglycaemia or “failure to thrive” in the presence of a metabolic acidosis;
- IV: unexplained single or multi-organ dysfunction or fulminant failure (including, but not limited to, neuropathies, myopathies, hepatopathy, pancreatic and/or bone marrow failure);
- V: refractory or atypical seizures, developmental delays or cognitive regression, or progressive encephalopathy or progressive encephalomyopathy;
- VI: cardiomyopathy and/or cardiac arrythmias;
- VII: rapid hearing or painless visual loss or ptosis;
- VIII: stroke-like episodes or nonvasculitic strokes;
- IX: ataxia, encephalopathy, seizures, muscle fatigue or weakness;
- X: external ophthalmoplegia;
- XI: hearing loss, diabetes, unexplained short stature, or endocrinopathy;
- XII: family history of mitochondrial disease, or any of the above; and

e) the service is not a service associated with a service to which item 73358, 73359 or 73456 applies.

Applicable only once per lifetime

**Supplementary Material 2**

**
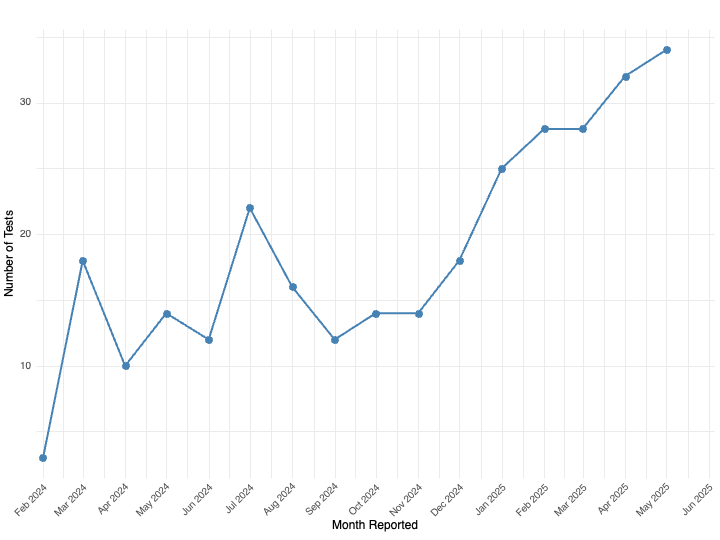
**

Supplementary Material 2: **Monthly uptake of genomic testing**: The number of tests reported in each month after the testing became available in November 2023, showing a gradual increase in uptake as time progresses.

References

1. Department of Health and Aged Care. Item 73456. Medicare Benefits Schedule. Updated November 1, 2023. Accessed August 12, 2025. <https://www9.health.gov.au/mbs/fullDisplay.cfm?type=item&q=73456&qt=item&criteria=73456>

2. Department of Health and Aged Care. Item 73457. Medicare Benefits Schedule. Updated November 1, 2023. Accessed August 12, 2025. <https://www9.health.gov.au/mbs/fullDisplay.cfm?type=item&q=73457>
